# High performance and remarkable cyclic stability of a nanostructured RGO–CNT-WO_3_ supercapacitor electrode

**DOI:** 10.1039/d1ra08413e

**Published:** 2022-04-11

**Authors:** Farah Nasreen, Abdul Waheed Anwar, Abdul Majeed, Muhammad Ashfaq Ahmad, Usman Ilyas, Furqan Ahmad

**Affiliations:** Department of Physics, University of Engineering and Technology Lahore 548900 Pakistan abdulwaheedanwar@uet.edu.pk; Institute of Metal Research, Chinese Academy of Sciences Shenyang China; COMSATS Institute of Information and Technology Lahore 54000 Pakistan; Department of Metallurgical Engineering, University of Engineering and Technology Lahore 548900 Pakistan

## Abstract

One of the most pressing concerns in today's power networks is ensuring that consumers (both home and industrial) have access to efficient and long-lasting economic energy. Due to improved power accessibility and high specific capacitance without deterioration over long working times, supercapacitor-based energy storage systems can be a viable solution to this problem. So, here, tungsten trioxide (WO_3_) nanocomposites containing reduced graphene oxide and carbon nanotubes *i.e.* (RGO-WO_3_), (CNT-WO_3_), and (RGO–CNT-WO_3_), as well as pure WO_3_ nanostructures as electrode materials, were synthesized using a simple hydrothermal process. The monoclinic phase of WO_3_ with high diffraction peaks is visible in X-ray diffraction analysis, indicating good crystallinity of all electrode materials. Nanoflowers of WO_3_ were well-decorated on the RGO/CNTs conductive network in SEM micrographs. In a three-electrode system, the specific capacitance of the RGO–CNT-WO_3_ electrode is 691.38 F g^−1^ at 5 mV s^−1^ and 633.3 F g^−1^ at 2 A g^−1^, which is significantly higher than that of pure WO_3_ and other binary electrodes. Furthermore, at 2 A g^−1^, it achieves a coulombic efficiency of 98.4%. After 5000 cycles, RGO–CNT-WO_3_ retains 89.09% of its capacitance at 1000 mV s^−1^, indicating a promising rate capability and good cycling stability performance.

## Abstract 2

One of the most pressing concerns in today's power networks is ensuring that consumers (both home and industrial) have access to efficient and long-lasting economic energy.

## Graphical abstract

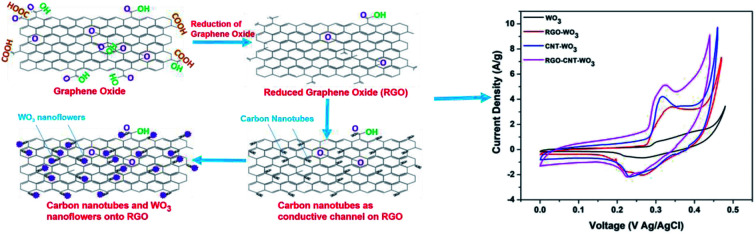

## 1. Introduction

The development of energy storage systems (ESSs) is crucial for tackling climate change and the finite availability of fossil fuels, as well as for storing solar and wind energy efficiently. One of the most difficult challenges now facing academics is to build highly efficient, low-cost, and environmentally friendly ESS devices. With the rapid rise of the portable electronic device industry and the development of hybrid electric vehicles, the demand for high-energy and high-power density energy storage technologies has surged. Even at increased power densities, the stored energy should be able to be released smoothly and distributed. Among these ESSs, supercapacitors are one of the most important technologies for energy storage applications because they can give better power and energy densities than batteries and ordinary dielectric capacitors.^1–4^ A number of materials have already been studied as supercapacitor electrode materials. Amongst all, RuO_2_ and IrO_2_ are good supercapacitor electrode materials because they have a high specific capacitance value and a high cycle capacity, which has sparked increased interest in this subject. However, their high cost and toxicity limit their practical application. One of the most active research subjects in electrochemistry is the creation of alternative economical and environmentally acceptable electrode materials with good performance. Many researchers are now working on alternate materials to IrO_2_ and RuO_2_. One of the most enticing alternatives to RuO_2_ and IrO_2_ may be WO_3_ owing to its electrochemical redox characteristics, environmental friendliness and low cost.^5–7^ Tungsten trioxide (WO_3_) is a well-known wide band gap n-type semiconductor with a variety of unique features and many crystal forms that are suited for the intercalation of tiny cations such as H^+^. It's been explored extensively as a possible material for a variety of applications which includes solar energy devices, semiconductor gas sensors, photocatalysts, electrode materials for secondary batteries and supercapacitors, field-emission devices. As a supercapacitor electrode, the capacitive characteristics of tungsten oxides *e.g.* mesoporous tungsten oxide, nanostructured tungsten trioxide, amorphous tungsten oxide and others have been studied.^8–11^ Xu He *et al.* developed and synthesized tungsten trioxide microspheres electrode materials with a specific capacitance of 488.78 F g^−1^.^12^ M. Ashraf *et al.* presented an asymmetric supercapacitor (HRG/m-WO_3_ ASC) made of monoclinic tungsten oxide (m-WO_3_) nanoplates for the negative electrode and highly reduced graphene oxide (HRG) for the positive electrode and exhibited a specific capacitance of 389 F g^−1^ at a current density of 0.5 A g^−1^.^13^ Nevertheless, the main disadvantages of tungsten oxide pseudocapacitors are their limited electrical conductivity and weak rate performance. Supercapacitor resistance should be reduced to increase rate capability. However, tungsten oxide preparations with diverse morphologies have received a lot of attention in terms of capacitive characteristics. The majority of investigations, on the other hand, focused on WO_3_ nanostructures produced from nanostructures, nanorods, or films. Until now, the capacitive characteristics of WO_3_ and carbon composites have received little attention in research.^14^ Concerning greater surface area, environmental friendliness, linked pore structure, high electrical conductivity and pore size regulation, carbon materials with varied micro textures are considered the major choice for supercapacitors.^15–18^ When WO_3_ is mixed with carbon/graphene sheets that are extremely conductive, its conductivity greatly enhanced, resulting in good capacitive materials with excellent system conductivity. A 1D multi-walled CNTs (MWCNTs) and reduced graphene oxide interwoven network also enhance electrical conductivity while also providing greater electrochemically active surface area and efficient routes for both electrons and ions in a hybrid electrode.^19^ The electrical double layer capacitor (EDLC) and pseudocapacitive processes in composites of WO_3_ and RGO/MWCNTs are of particular interest because they contain a dual charge storage mechanism.^20^ In addition, chemical vapour deposition, sol–gel, hydrothermal technique, and other methods have been used to synthesize WO_3_ nanostructures in the current findings. Among these, the hydrothermal approach was deemed superior because it allows the preparation of WO_3_ nanoflowers in a mild, well-controlled, and cost-effective manner,^21^ which was ideal for our experiment. In the present work, a facile hydrothermal procedure is demonstrated to prepare monoclinic tungsten trioxide combined with carbon nanotubes and reduced graphene oxide *i.e.* RGO–CNT-WO_3_ composite electrode. The electrochemical experiments revealed that the RGO–CNT-WO_3_ composite electrode has better capacitive properties than the WO_3_, RGO-WO_3_, CNT-WO_3_ electrode, with greater reversible charging/discharging capabilities and larger capacitance values along with high cyclic stability. The flower-like architectures of WO_3_ are very well assembled on reduced graphene sheets that are connected to CNTs with strong interfacial contact in RGO/CNT based tungsten trioxide nanocomposites, which ultimately provides the fastest electron transportation and thus enhances the performance of electrode.

## 2. Experimental section

The tungsten precursor was sodium tungstate dihydrate (Na_2_WO_4_·2H_2_O). The pH was adjusted with hydrochloric acid (HCl). DI water was used to dissolve the precursor, dilute the acid to the appropriate molarity, and wash the finished product. Ethanol was also used to remove any contaminants from the finished product. Nickel foam was employed as the substrate for slurry casting the as-prepared powder. The samples were slurry cast onto the substrate using PVDF, Nafion, and carbon black. Sodium dodecyl benzenesulfonate (SDBS) was used to make CNTs solution in DI water. All of the reagents were acquired from Sigma Aldrich Co. LLC and utilized without additional purification. Nickel (Ni) foam was purchased from Winfay Group Co. Ltd, China.

### 2.1 Synthesis of WO_3_ nanostructures

**Fig. 1:**
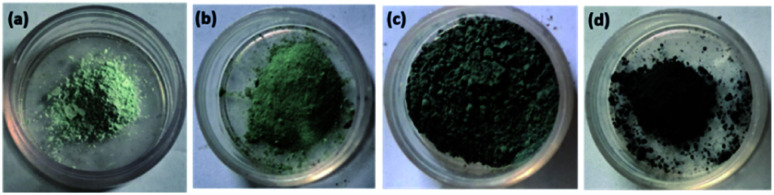
Digital photographs of (a) WO_3_ (b) RGO-WO_3_ (c) CNT-WO_3_ (d) RGO–CNT-WO_3_.

First of all, 2 g of Na_2_WO_4_·2H_2_O was dissolved in 50 ml DI water followed by the addition of 3 M HCl (*i.e.* 12.5 ml HCl in 50 ml DI water) until the pH reached 1. After that, 3.06 g of oxalic acid and 4 g of (NH_4_)_2_SO_4_ were added. The mixture was transferred to a stainless steel autoclave lined with Teflon and holding 150 ml. The autoclave was sealed and placed in an oven at 180 °C for 16 hours, after which it was allowed to cool naturally. The precipitate was filtered and washed with DI water and ethanol multiple times before being vacuum dried for 12 hours at 80 °C (light grey colour Fig. 1a).

### 2.2 Synthesis of RGO-WO_3_ nanostructures

To begin, a 40 ml (1 mg/1 ml) graphene oxide solution was prepared using sonication and centrifugation. The RGO-WO_3_ nanocomposite was then made by dissolving 2 g of Na_2_WO_4_·2H_2_O in 30 ml of deionized water and stirring it for 30 minutes with a magnetic stirrer. Then, drop by drop, 3 M HCl was added until the pH reached up to 1.3.06 g of oxalic acid and (NH_4_)_2_SO_4_ were added after that. The solution was then diluted with 50 ml of deionized water. After that, the GO solution was gently added using sonication for 45 minutes. The solution was then put down into a Teflon-lined stainless steel autoclave and heated for 20 hours at 180 °C. The RGO-WO_3_ precipitate was filtered and washed multiple times with acetone and DI water and placed further in a vacuum oven to dry at 80 °C for 12 h (Fig. 1b).

### 2.3 Synthesis of CNT-WO_3_ nanostructures

First, 10 mg ml^−1^ MWCNTs were ultrasonically dispersed in 5 mg ml^−1^ sodium dodecyl benzenesulfonate (SDBS) for 8 hours. The CNT-WO_3_ nanocomposite was then synthesized by dissolving 2 g of Na_2_WO_4_·2H_2_O in 30 ml of deionized water and stirring it for 30 minutes with a magnetic stirrer. Then, drop by drop, 3 M HCl was added until the pH reached 1.3.06 g of oxalic acid and 4 g of (NH_4_)_2_SO_4_ were added after that. The solution was then diluted with 50 ml of deionized water. After that, the CNT solution was gently added using sonication for 45 minutes. The solution was then put into a Teflon-lined stainless steel autoclave and heated for 20 hours at 180 °C. The CNT-WO_3_ precipitates were obtained through filtration and washing with acetone and DI water multiple times and placed further in a vacuum oven to dry at 80 °C for 12 h shown in Fig. 1c.

### 2.4 Synthesis of RGO–CNT-WO_3_ nanostructures

**Fig. 2:**
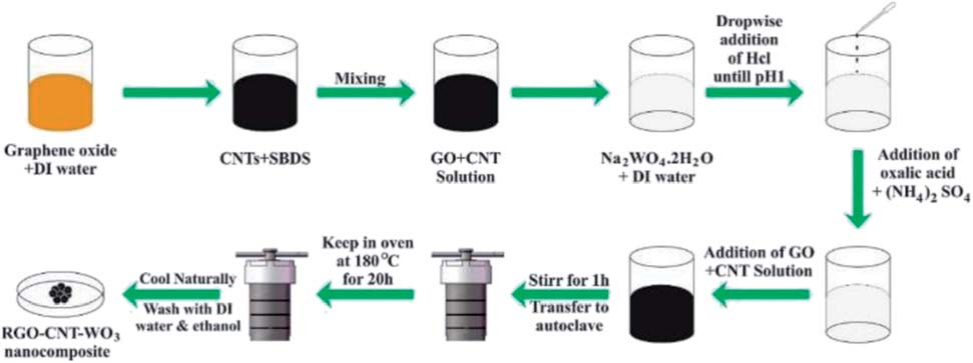
Schematic diagram of the synthesis of RGO–CNT-WO_3_ through hydrothermal method.

By sonication for 9 hours, 10 mg ml^−1^ MWCNTs were disseminated in 5 mg ml^−1^ SDBS. Then, to make GO/CNT dispersion, 40 mg ml^−1^ graphene oxide solution was mixed thoroughly with CNTs solution. The RGO–CNT-WO_3_ nanocomposite was then synthesized by dissolving 2 g of Na_2_WO_4_·2H_2_O in 30 ml of deionized water and stirring it for 30 minutes with a magnetic stirrer. Then, drop by drop, 3 M HCl was added until the pH reached 1. Then, 3.06 g of oxalic acid and 4 g of (NH_4_)_2_SO_4_ were added after that. The solution was then diluted with 50 ml of deionized water. After that, the CNT solution was gently added using sonication for 45 minutes. The solution was then positioned into a Teflon-lined stainless steel autoclave and heated for 20 hours at 180 °C. RGO–CNT-WO_3_ precipitates were obtained by filtration and washing with acetone and DI water multiple times before being dried for 12 hours in a vacuum oven at 80 °C. The final black product is exhibited in Fig. 1d. The Schematic diagram of the synthesis of RGO–CNT-WO_3_ through hydrothermal method has been displayed in Fig. 2.

### 2.5 Structural characterization

The crystal structure was studied through X-ray diffraction (XRD; Rigaku Diffractometer) using Cu Kα radiation with a wavelength of (*λ* = 1.5416 Å) and a temperature range of 5° to 60°. Raman Spectroscopy (Jobin Yvon HR800) was also used to do the Raman analysis, which used a He–Ne laser with a wavelength of 632.8 nm. Scanning Electron Microscopy was employed to investigate the morphology of nanocomposites (Nova NanoSEM 430, 10 kV).

### 2.6 Electrochemical characterization and fabrication of electrodes

Under a three-electrode cell configuration in 3 M KOH at ambient temperature, cyclic voltammetry (CV), galvanostatic charge–discharge (GCD), electrochemical impedance spectroscopy (EIS), and cyclic stability tests of composite electrodes were investigated. As working, counter, and reference electrodes, the as-prepared composite electrodes, platinum wire, and Ag/AgCl were employed respectively. On an electrochemical workstation (Gamry Reference 3000 Instrument, USA), all electrochemical properties were recorded. To make the working electrodes, the nickel sponge was cleaned with acetone, 2 M HCl, ethanol, and DI water many times for 15 minutes each. After that, it is dried entirely at 90 °C for 12 hours. The nickel sponge was sliced into (1 × 1 cm^2^) dimensions after drying. In NMP solution, the electrode active material, polyvinylidene flouride (PVDF), and acetylene black were mixed in 80 : 10 : 10 ratio to produce a homogenous slurry. Finally, this paste was spread onto sponge to make composite electrodes, which were then dried for 8 hours at 90 °C. The difference between loaded and unloaded nickel foam was used to compute the loading density of active materials for composite electrodes. The loading density of active materials for all electrodes was 1 mg cm^−2^. Using an Ag/AgCl reference electrode, CV experiments were conducted in the 0–0.48 voltage range at 5, 10, 20, 40 and 50 mV s^−1^. GCD tests were performed at densities of 2, 4, 6, and 8 A g^−1^. EIS study was performed with a 10 mV AC voltage amplitude and a frequency range of 1 Hz to 10^5^ Hz. Eqn (1) and (2) are used to calculate specific capacitance (*C*_s_) from CVs and galvanostatic discharging curves respectively;1
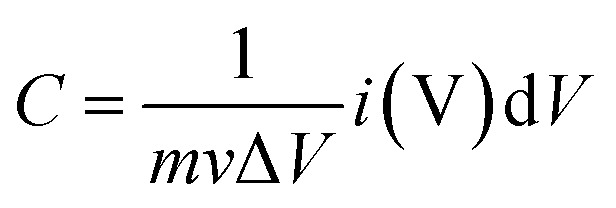
2
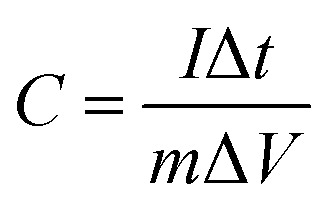
where, *i* (V) is voltammetric current, *m* (g) represents the mass of active materials, Δ*V* (V) is the potential window, *ν* (mV s^−1^) is the scan rate, *I* (A) is applied current while Δ*t* (s) is total discharging time.^22,23^

The coulombic efficiency of an electrode can be computed by using the following relation3
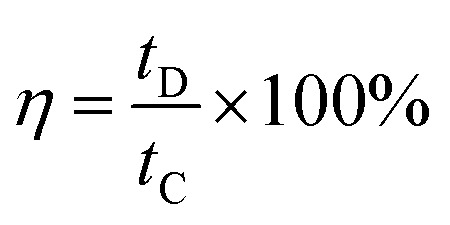
while *t*_D_ and *t*_C_ are charging and discharging time respectively.^24^

## 3. Results and discussion

### 3.1 Physical characterizations

The crystallinity of WO_3_, RGO-WO_3_, CNT-WO_3_, RGO-CNT-WO_3_ materials was studied using X-ray diffraction (XRD, Rigaku diffractometer) with Cu Kα radiation (*λ* = 1.5416 Å). Scanning electron microscopy (SEM, Hitachi S4800, operating voltage 10 kV) was used to examine the morphologies of the produced materials. Raman spectrometer (Jobin Yvon HR 800) equipped with He–Ne laser was used to record Raman spectra at an excitation source of 632.8 nm wavelength.

#### 3.1.1 XRD analysis

**Fig. 3:**
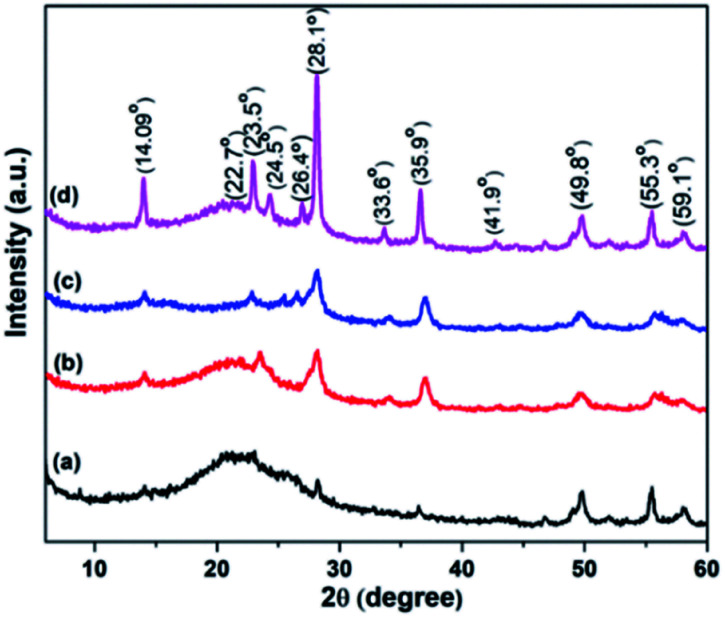
XRD pattern of (a) WO_3_ (b) RGO-WO_3_ (c) CNT-WO_3_ (d) RGO–CNT-WO_3_ nanostructures.

To determine the crystalline nature of samples, X-ray diffraction is employed. The range to analyse spectra was 5° to 60°. Fig. 3 manifests the XRD spectra of WO_3_, RGO-WO_3_, CNT-WO_3_, RGO–CNT-WO_3_ nanostructures. All assigned peaks referred to monoclinic phase of tungsten trioxide well matched with JCPDS card no. 83-9950 having space group *P*2_1/*n*_ (*a* = 7.297 Å, *b* = 7.539 Å, *c* = 7.689 Å). No impurity peak is found in this pattern. The favourable oriented growth is observed in crystallite planes (100), (002), (200), (112), (220), (122), (222), (232), (420), (224), (402) having value of 2*θ* as 14.09°, 22.7°, 24.5°, 28.1°, 33.6°, 35.9°, 41.9°, 49.8°, 55.3°, 59.1°, 63.7°.^25–27^ The high crystallinity helps to improve the overall electrochemical performance of synthesized electrodes.^28^ A peak observed at 23.5° specified to the successful conversion of graphene oxide into reduced graphene oxide through hydrothermal route. The XRD patterns of composites did not show any peak of GO at 10°, indicating that the GO reduction was done properly throughout the composite preparation. The presence of CNTs is confirmed by the peak located at 26.4°.^29^

#### 3.1.2 SEM analysis

**Fig. 4:**
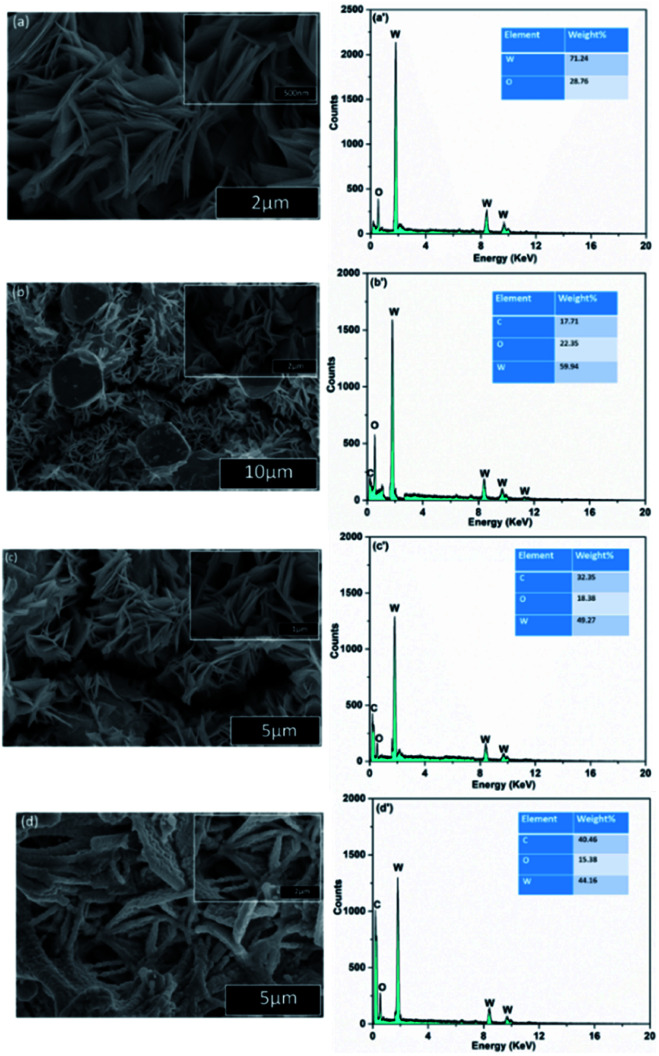
SEM images of (a) WO_3_ (b) RGO-WO_3_ (c) CNT-WO_3_ (d) RGO–CNT-WO_3_ nanostructures and the corresponding EDS graphs of (a′) WO_3_ (b′) RGO-WO_3_ (c′) CNT-WO_3_ (d′) RGO–CNT-WO_3_.

The morphological characteristics of WO_3_, RGO-WO_3_, CNT-WO_3_, RGO–CNT-WO_3_ were studied by scanning electron microscopy. Fig. 4 shows that WO_3_ nanocomposites have uniform 3 dimensional architectures having flowerlike shape. In addition, highly magnified images endorse that these architectures are composed of many sheet-like petals and no breakage or collapse is observed in these petals. In RGO/CNT based tungsten trioxide nanocomposites, these flower like architectures of WO_3_ are assembled very well on reduced graphene sheets which are connected to CNTs having strong interfacial contact which ultimately provides the fastest electron transport and hence enhances the performance of electrode.^29^

The pores in RGO/CNTs network provide vast channels for ion-transportation and inter-connected sheet works as conduction skeleton for electron transportation. This unique feature also provides enhanced contact area with electrolytic ions which results in path length reduction thus leads to promotion of fast electrons and ion transportation. This will overall improve the electrochemical performance.^30^ The corresponding EDS spectra of all nanocomposites, displayed in Fig. 4a′–d′ indicate the presence of C, O and W elements without any other impurities.

#### 3.1.3 Raman analysis

**Fig. 5:**
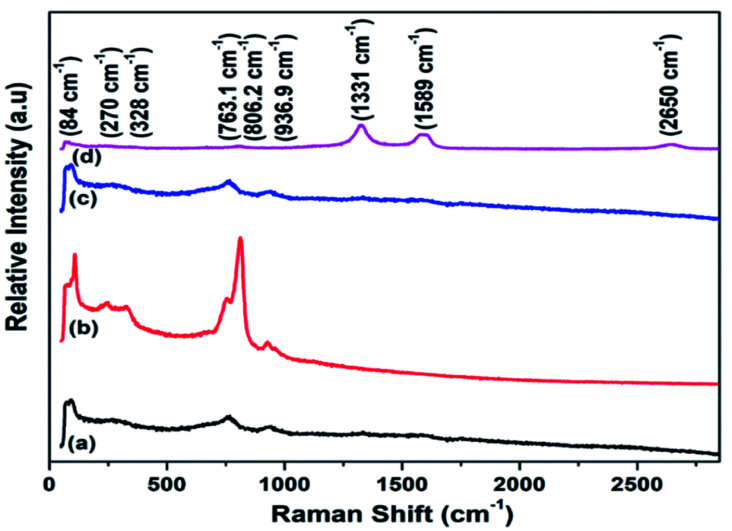
Raman spectra of (a) WO_3_ (b) RGO-WO_3_ (c) CNT-WO_3_ (d) RGO–CNT-WO_3_ nanostructures.

The phonons, defects and electron–electron/phonon–electron interactions in different materials are studied through Raman analysis. Fig. 5 presents the Raman spectra of WO_3_, RGO-WO_3_, CNT-WO_3_, RGO–CNT-WO_3_ nanostructures.

As depicted in Fig. 5, there are six modes (Raman active) for tungsten trioxide (WO_3_). A G peak, D peak and 2D bands for RGO-CNTs are located at 1331 cm^−1^, 1589 cm^−1^ and 2650 cm^−1^ respectively. A D-band referred to defects present in carbon nanostructures whereas scattering of sp^2^ carbon atoms results in G-peak. Also, 2D bands are created as a result of dual resonance processes. The peak located at 936.9 cm^−1^ attributes to W=O bond stretching, whereas stretching vibration of oxygen (O–W–O) is related to the peaks 763.1 cm^−1^ and 806.2 cm^−1^ respectively. The bending vibration of (W–O–W) occurs and results in a peak at 264 cm^−1^ while peak located at 328 cm^−1^ correlates to *v* (W–OH_2_) vibrations. Additionally, a pair of peaks are found at 84 cm^−1^ and 270 cm^−1^. Also, in addition, two low-intensity peaks referred to D & G bands, representing the presence of RGO-CNTs in CNT-WO_3_, RGO-WO_3_ and RGO–CNT-WO_3_ nanostructures.^31–34^

### 3.2 Electrochemical characterizations

To prepare the working electrodes, the active materials (80 wt%), acetylene black (10 wt%) and PVDF (solvent is NMP) are mixed and pressed on a nickel foam and finally dried in an oven at a temperature of 120 °C for 10 hours. The cyclic voltammetry (CV), galvanostatic charge discharge (GCD), cyclic stability and electrochemical impedance spectroscopy (EIS) were employed to study the electrochemical features of as-prepared electrodes in a three-electrode cell configuration in 3 M KOH electrolytic solution while this cell configuration contains a Pt wire acting as counter electrode, as-prepared electrodes as working electrode and Ag/AgCl electrode worked as reference electrode. A gold wire is used as current collector. These measurements were taken in a potential range 0–0.48 V at various scan rates. Different current densities (2–8 A g^−1^) were employed to record GCD curves. The EIS spectra was taken for frequency range (1–105 Hz) using 10 mV AC perturbation. Cyclic voltammetry (CV) was employed to inspect stability performance of (RGO–CNT-WO_3_) hybrid electrode. The CV cycles were run for 5000 cycles at a scan rate of 1000 mV s^−1^ and two CV curves were recorded at scan rate 5 mV s^−1^ initially and after 5000th cycle and then capacitance retention of prepared electrode was calculated.

#### 3.2.1 CV analysis

**Fig. 6:**
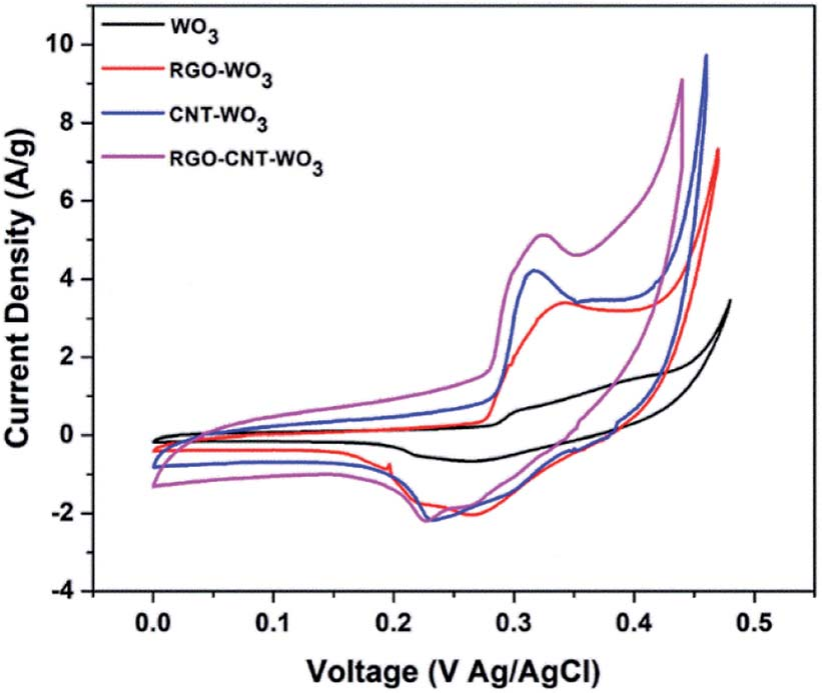
Comparison of CV curves of WO_3_, RGO-WO_3_, CNT-WO_3,_ RGO–CNT-WO_3_ electrodes at a scan rate of 5 mV s^−1^.

The super-capacitive behaviour of as-synthesized electrodes was studied through CV analysis. Fig. 6 manifests the comparison of CV curves of WO_3_, RGO-WO_3_, CNT-WO_3_ and RGO–CNT-WO_3_ electrodes at a scan rate of 5 mV s^−1^. A couple of redox peaks are traced in all CV curves owing to psudocapacitive behaviour arises due to electrochemical reactions. It has been distinctly seen that RGO–CNT-WO_3_ electrode displays greater integrated area in comparison to other electrodes implying the fast interaction among RGO/CNT and tungsten trioxide nanostructures which speed up the electro-chemical activity, hence the effective and fast transfer of charges.^35^

**Fig. 7:**
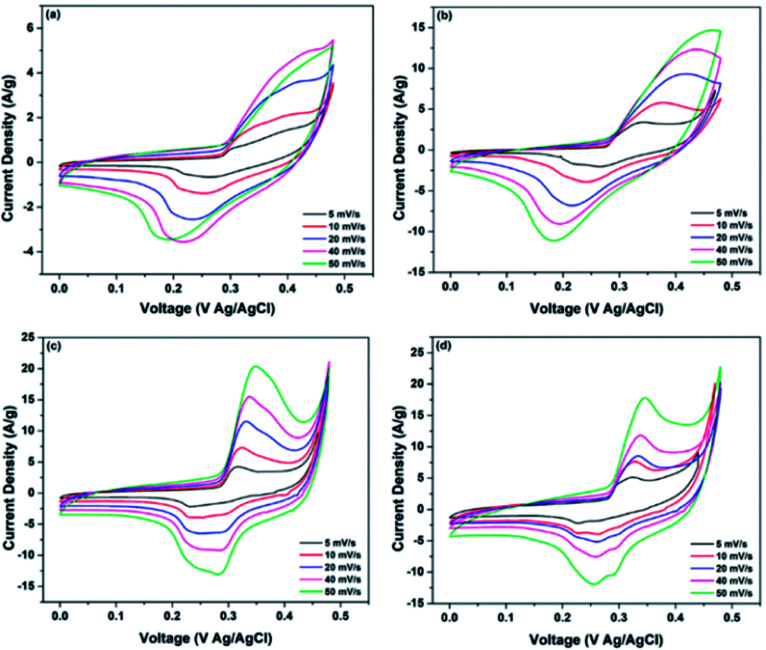
Comparison of CV curves of (a) WO_3_, (b) RGO-WO_3_, (c) CNT-WO_3_, (d) RGO–CNT-WO_3_ electrodes at various scan rates.

Eqn (1) was used to compute the specific capacitance of each electrode. Among all the electrodes, RGO–CNT-WO_3_ composite owns higher capacitance of 691.38 F g^−1^ as compared to other electrodes (Table 1). The higher accessible surface area facilitates faster ion adsorption which leads to efficient transportation of charges and ion intercalation^36^ resulting in higher specific capacitance of RGO–CNT-WO_3_. The supercapacitive performance of WO_3_, RGO-WO_3_, CNT-WO_3_, RGO–CNT-WO_3_ from 5-50 mV s^−1^ are displayed in Fig. 7.

**Table 1:** Specific capacitances of WO_3_, RGO-WO_3_, CNT-WO_3_, RGO–CNT-WO_3_ electrodes at different scan rates

Sample	Specific capacitance (F g^−1^)				
	5 mV s^−1^	10 mV s^−1^	20 mV s^−1^	30 mV s^−1^	50 mV s^−1^
WO_3_	212.93	156.3	129.4	120.6	67.32
RGO-WO_3_	497.6	387.91	312.8	286.3	199.08
CNT-WO_3_	567.99	481.97	381.07	334.3	247.94
RGO–CNT-WO_3_	691.38	627.03	344.03	304.6	253.09

As expected, the increase in scan rate resulted in increased current density and integrated area with nearly little change in shape of CV curves which reflects the higher reversibility and stability of all electrodes. Nevertheless, as presented in Fig. 7d, even at higher scan rates of 50 mV s^−1^, there is no remarkable change in shapes of CV curves which indicates behaviour close to ideal supercapacitor and high rate performance as compared to other electrodes.

**Fig. 8:**
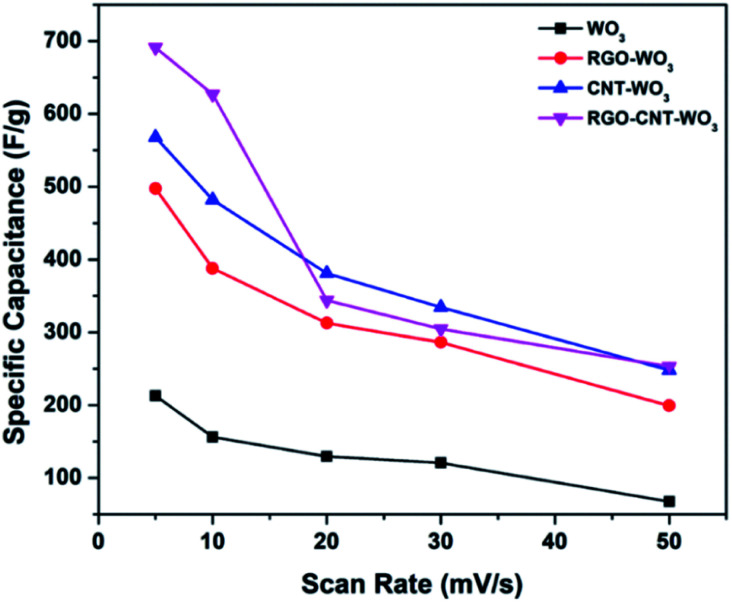
The variation of specific capacitance with scan rates of WO_3_, RGO-WO_3_, CNT-WO_3_, RGO–CNT-WO_3_ electrodes at various scan rates.

The specific capacitance variation with scan rates is depicted in Fig. 8. It can be clearly seen that the increase in scan rate reduces specific capacitance because the redox reactions occur only onto the surface as inner electroactive sites for ion intercalation are not available completely and *vice versa*.

#### 3.2.2 GCD analysis

**Fig. 9:**
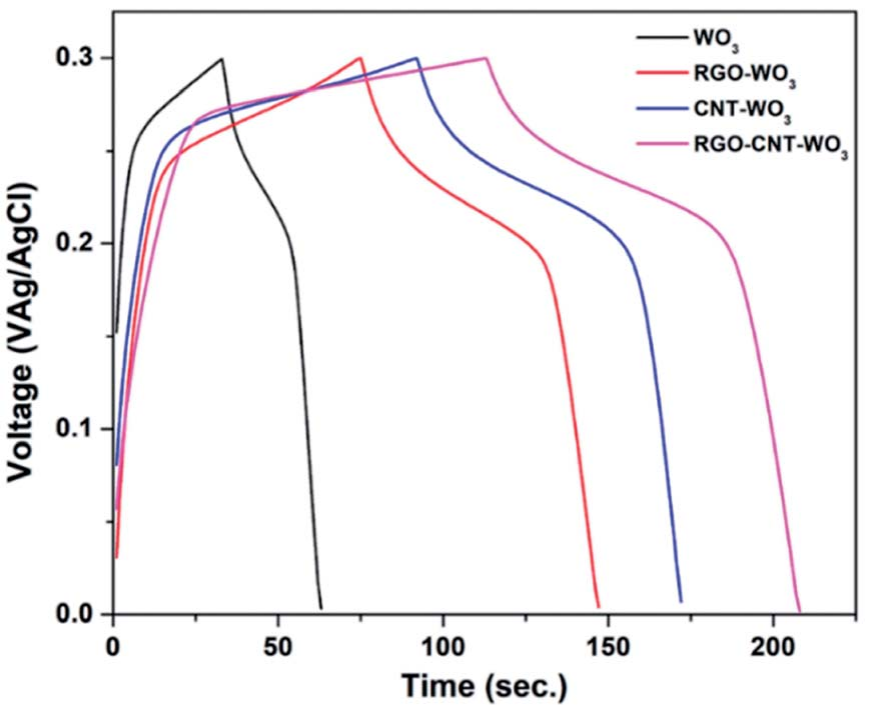
Comparison of GCD curves of WO_3_, RGO-WO_3_, CNT-WO_3_, RGO–CNT-WO_3_ electrodes at a current density of 2 A g^−1^.

Galvanostatic charge–discharge analysis is another reliable method for enquiry of electrochemical performance of all electrodes. The GCD curves of WO_3_, RGO-WO_3_, CNT-WO_3_ and RGO–CNT-WO_3_ electrodes at 2 A g^−1^ in 3 M KOH electrolytic solution are shown in Fig. 9. All GCD curves reveal typical triangular discharge plateau which are in good symmetry, exhibiting pseudocapacitive behaviour. These GCD curves also display redox peaks which is in accordance with CV curves. Eqn (2) has been used to calculate specific capacitance of all electrodes and the obtained values are 199.7 F g^−1^, 480.6 F g^−1^, 533.3 F g^−1^, 633.3 F g^−1^ for WO_3_, RGO-WO_3_, CNT-WO_3_ and RGO–CNT-WO_3_ respectively at 2 A g^−1^. In agreement to CV curve, RGO–CNT-WO_3_ has longer discharge time, thus exhibiting higher specific capacitance (*C*_s_) of 633.3 F g^−1^ in comparison of WO_3_, RGO-WO_3_ and CNT-WO_3_ electrodes.

Such greater performance of RGO–CNT-WO_3_ can be credited to (i) the short ion diffusion path and facilitates rapid electron transport which is produced as a result of sufficient interfacial contact amongst RGO/CNT nanosheets and WO_3_ nanostructures (ii) WO_3_ nanoflowers permit reversible and instantaneous oxidation–reduction reactions to enhance the capacitance (iii) the production of electroactive sites increased due to unique hybrid nanostructures.^37^

The RGO–CNT-WO_3_ electrode possesses specific capacitances of 633.3, 562.6, 429 & 340.7 F g^−1^ at 2, 4, 6 and 8 A g^−1^ respectively [Table 2]. Distinctly, even at a high current density of 8 A g^−1^, the capacitance of the RGO–CNT-WO_3_ electrode remains as high as 346.7 F g^−1^.

**Table 2:** Specific capacitances of WO_3_, RGO-WO_3_, CNT-WO_3_, RGO–CNT-WO_3_ electrodes at various current densities

Sample	Specific capacitance (F g^−1^)			
	2 A g^−1^	4 A g^−1^	6 A g^−1^	8 A g^−1^
WO_3_	199.7	160.27	72.4	52.8
RGO-WO_3_	480.6	400.5	238.6	211.47
CNT-WO_3_	533.3	425.3	361	318.4
RGO–CNT-WO_3_	633.3	562.7	429	346.7

**Fig. 10:**
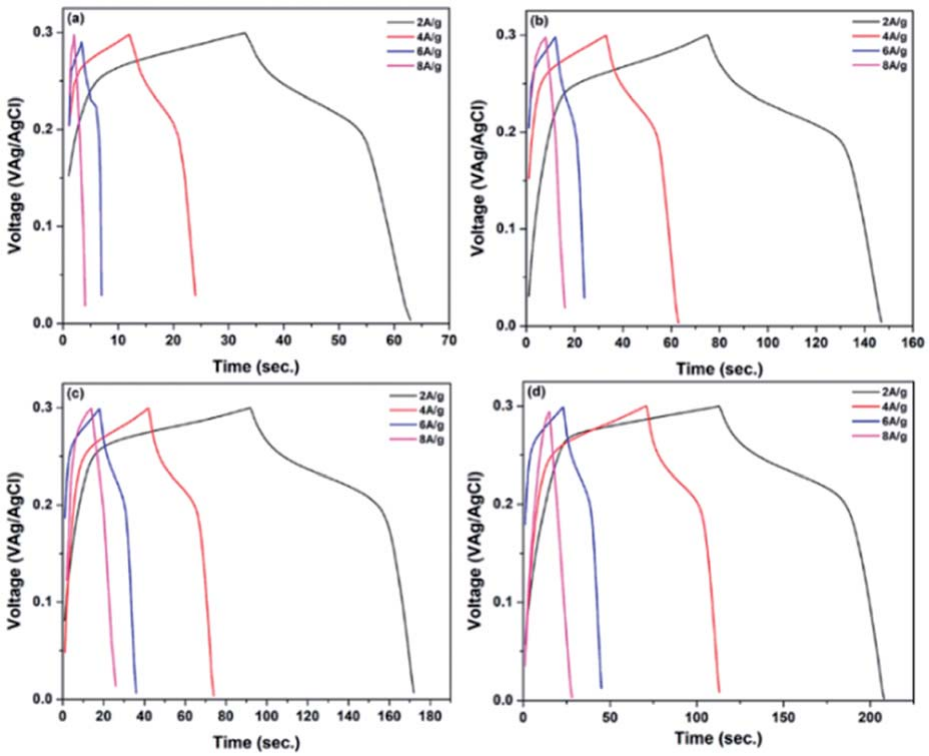
GCD curves of (a) WO_3_ (b) RGO-WO_3_ (c) CNT-WO_3_ (d) RGO–CNT-WO_3_ electrodes at different current densities.

**Fig. 11:**
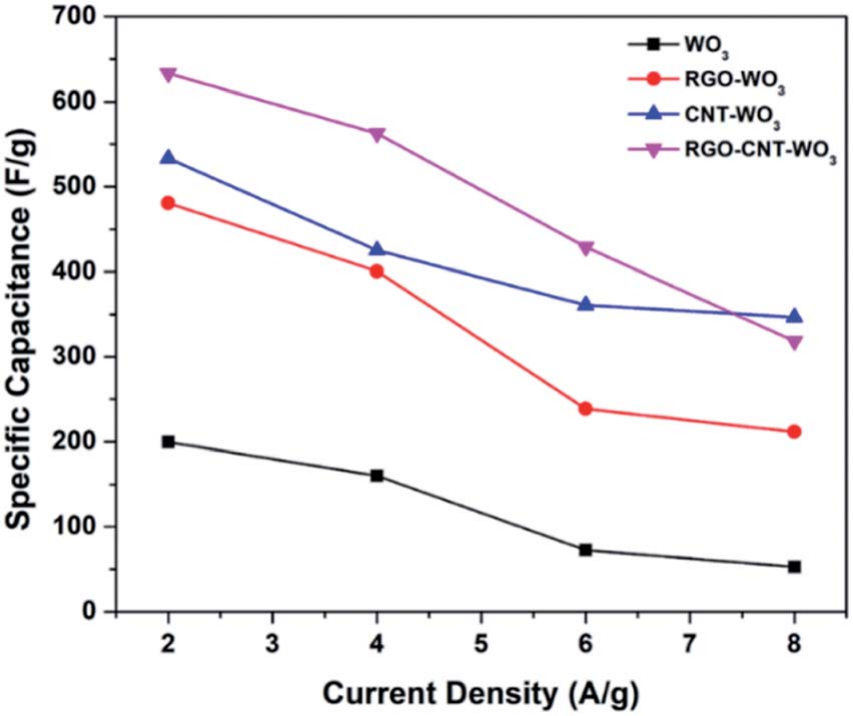
Relationship of specific capacitance as a function of current density of WO_3_, RGO-WO_3_, CNT-WO_3,_ RGO–CNT-WO_3_ electrodes.

To study the effect of higher current densities on specific capacitances, the GCD curves of each electrode at various current densities were obtained as represented in Fig. 10. It is clear that when the current density increases, the discharging time for all electrodes reduces. This is because at greater current densities, surface synergy between electrode and electrolyte is more likely to occur, resulting in a reduction in specific capacitance and a faster rate of discharge.^38,39^Fig. 11 illustrates the range of specific capacitance of RGO–CNT-WO_3_ electrodes with various current densities WO_3_, RGO-WO_3_, CNT-WO_3,_ RGO–CNT-WO_3_ electrodes with various current densities.

#### 3.2.3 EIS analysis

**Fig. 12:**
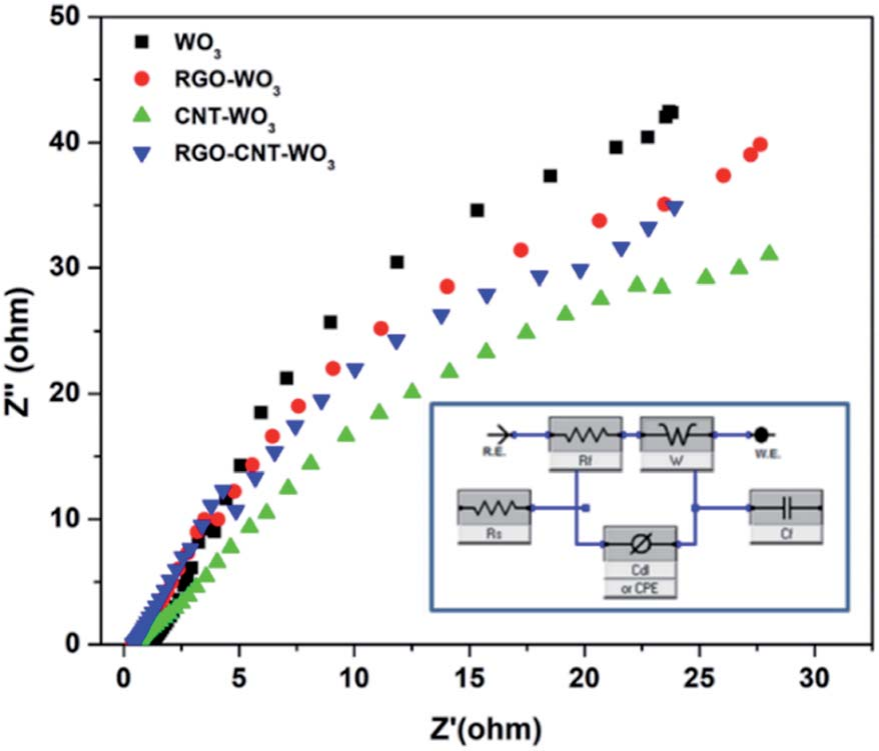
Comparison of Nyquist plots of WO_3_, RGO-WO_3_, CNT-WO_3_ and RGO–CNT-WO_3_ composite electrodes while inset shows an equivalent fitting circuit of a supercapacitor.

The capacitive and resistive behaviour of WO_3_, RGO-WO_3_, CNT-WO_3_, RGO-CNT-WO_3_ electrodes were explored using EIS. Fig. 12 illustrates the impedances of as-prepared electrodes in a 3 M KOH electrolytic solution over a frequency range of 1 Hz–100 kHz at 10 mV AC amplitude.

It is noticeable that on the real component *i.e.* the *Z*′ axis, the diameter of a semicircle in high frequency zone is associated to the *R*_ct_, indicating greater ion diffusion into electrode pores. In the high frequency band, the Nyquist plot of all electrodes displays no semicircle, indicating optimal super-capacitive behaviour.^29,40^ In comparison to other electrodes, the RGO–CNT-WO_3_ has a relatively low charge transfer resistance of 5.182 Ω. A vertical curve larger than 45° in the low-frequency zone suggests considerable super-capacitive activity with low diffusion resistance in the electrodes. The analogous circuit (inset) was used to investigate the produced impedance spectra, and the results are provided in Table 3, where *C*_PE_ stands for constant phase element. The RGO/CNT conductive network that governs fast electron transfer channels and WO_3_ nanoflowers that facilitate electrolyte penetration and reduce ion and electron diffusion routes are responsible for the improved electrochemical performance.^41^

**Table 3:** Different parameters obtained from fitting of EIS curves of WO_3_, RGO-WO_3_, CNT-WO_3_, RGO–CNT-WO_3_ electrodes

Sample	Series resistance, *R*_s_ (Ω)	Charge-transfer resistance, *R*_ct_ (Ω)	Constant phase element, *C*_PE_	Warburg impedance, *W* (Ω)
WO_3_	1.051	8.773	0.0045	661.6 × 10^−3^
pRGO-WO_3_	0.350	8.508	0.5004	652.3 × 10^−3^
CNT-WO_3_	0.542	9.354	0.0064	638.8 × 10^−3^
RGO–CNT-WO_3_	0.388	5.182	0.0052	597.7 × 10^−3^

#### 3.2.4 Coulombic efficiency

The coulombic efficiencies of all electrodes are calculated using eqn (3) to determine their reversibility of redox processes, and the results are reported in [Table 4].

**Table 4:** Coulombic efficiencies of WO_3_, RGO-WO_3_, CNT-WO_3_, RGO–CNT-WO_3_ electrodes at different current densities

Electrode materials	Coulombic efficiency *η* (%)			
	2 A g^−1^	4 A g^−1^	6 A g^−1^	8 A g^−1^
WO_3_	90.6	92.1	95.6	112.4
RGO-WO_3_	93.1	97.3	110	99.07
CNT-WO_3_	93.3	95.7	98.2	99.9
RGO–CNT-WO_3_	94.3	96.2	97.5	99.99

**Fig. 13:**
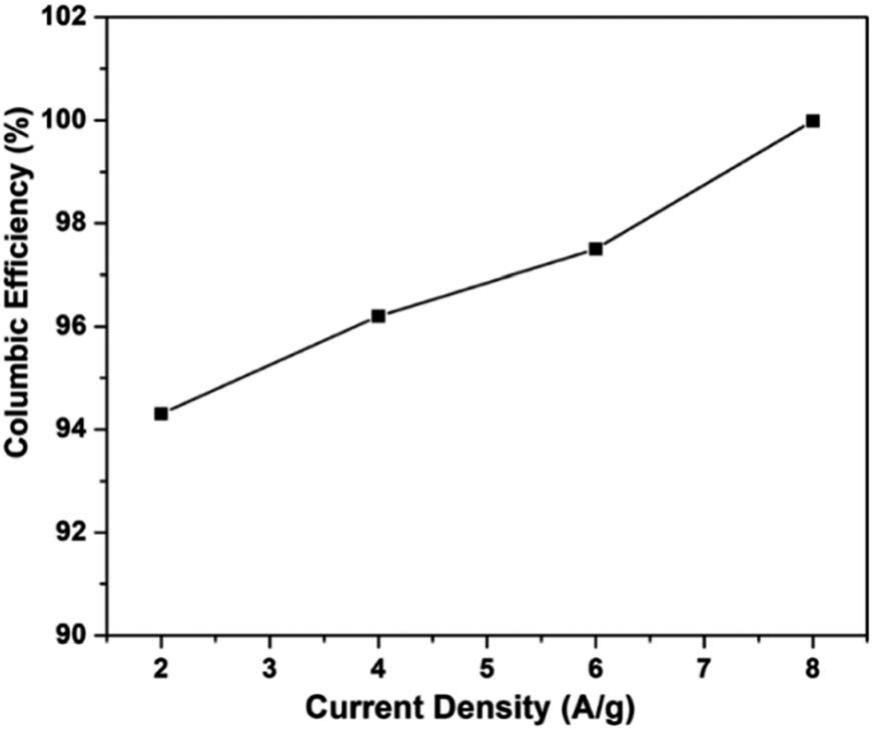
Coulombic efficiency as a function of current density of RGO–CNT-WO_3_ composite electrode.

It is noticed that the RGO–CNT-WO_3_ electrode has the highest coulombic efficiency of 94.3% that is more than 93.3%, 93.1% and 90.6% for WO_3_, RGO-WO_3_, CNT-WO_3_ accordingly at a current density of 2 A g^−1^. Fig. 13 shows the coulombic efficiency *vs.* current density, revealing the strong reversibility of the redox processes of the RGO–CNT-WO_3_ electrode. Furthermore, at a greater current density of 8 A g^−1^, the coulombic efficiency of WO_3_ exceeds 100%. At 6 A g^−1^, the RGO-WO_3_ electrode material achieves a coulombic efficiency of 110 percent. The increased (*η*) value (>100%) of RGO-WO_3_ at 6 A g^−1^ might be attributable to the breakdown of electrode material, which produces extra charges during the discharging cycle.^42^

#### 3.2.5 Cyclic stability

**Fig. 14:**
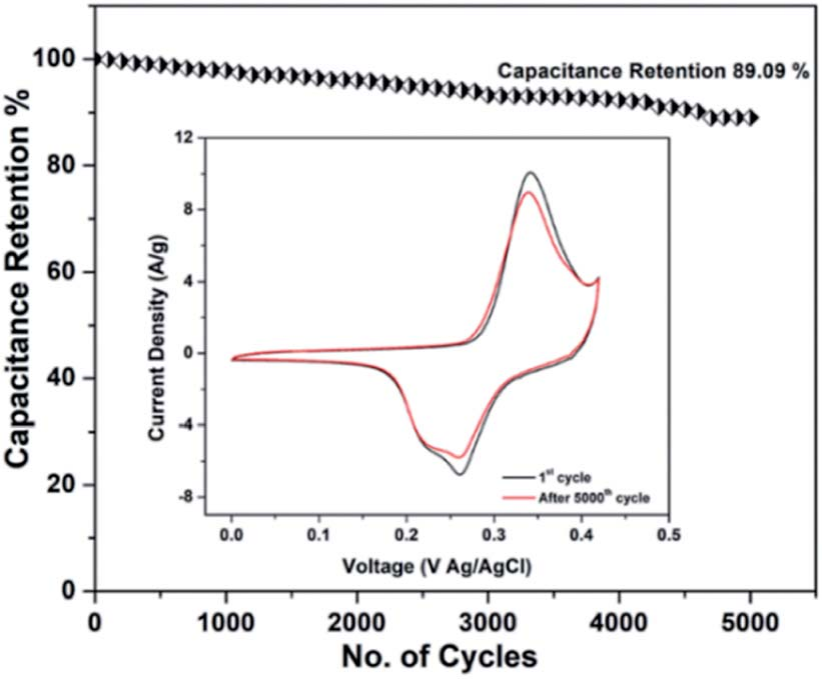
Cyclic stability of RGO–CNT-WO_3_ composite electrode at 1000 mV s^−1^ for 5000 cycles. The inset shows the CV curves obtained at 5 mV s^−1^ before and after 5000 cycles.

Fig. 14 shows the cyclic performance of the RGO–CNT-WO_3_ electrode over 5000 cycles, which is achieved by repeating the CV curves at 1000 mV s^−1^ between the potential 0–0.48 V. The RGO–CNT-WO_3_ electrode has a remarkable capacitance retention of 89.09 percent calculated through CV cycles obtained at 5 mV s^−1^ before and after 5000 cycles. These CV curves are shown in the inset of Fig. 14 at 5 mV s^−1^ for the first cycle and after the 5000th cycle.

The improved electro-chemical performance of RGO–CNT-WO_3_ can be accredited to tungsten trioxide and CNT/RGO nanostructures, which increase the electrolyte–electrode contact area and hence favour ion transport, resulting in a higher rate capability.^43^ These findings suggest that RGO–CNT-WO_3_ is a tremendous candidate for use in well-efficient energy storage devices. The comparison of electrochemical performance of (RGO-WO_3_), (CNT-WO_3_), and (RGO–CNT-WO_3_) with the reported literature was done and it is listed in Table 5. The results manifest that the prepared electrodes have capacitance and cyclic stability comparable to or higher than the others, suggesting the superior electrochemical performance. Also, to the best of our knowledge, RGO–CNT-WO_3_ electrode has not reported in the literature yet.

**Table 5:** Comparison of the electrochemical performance of all prepared electrodes with previously reported electrodes

Electrode materials	Synthesis method	Surface morphology	Electrolyte used	Specific capacitance (*C*_s_)	Capacitance retention	Ref.
Graphene nanosheets–tungsten oxide GNS–W	Facile solution approach	WO_3_ platelets	1 M H_2_SO_4_	143.6 F g^−1^		40
Feather duster-like CNT@WO_3_	Microwave heating method	WO_3_ nanosheet arrays	0.5 M H_2_SO_4_	496 F g^−1^ at 1 A g^−1^	96.3% after 8000 cycles	44
WO_3_·2H_2_O/bamboo charcoal	ϒ-Irradiation	Irregular forms	6 M KOH	391 F g^−1^ at 0.5 A g^−1^	82% retention after 10 000 cycles	45
HRG//m-WO_3_ ASC	Hydrothermal method	WO_3_ nanoplates	1 M H_2_SO_4_	389 F g^−1^ at 0.5 A g^−1^	92% after 5000 cycles	46
WO_3_-rGO hybrids	Hydrothermal method	Micro-rod like morphology	3 M KOH	801.6 F g^−1^ at 4 A g^−1^	75.7% after 5000 cycles	47
RGO–CNT-WO_3_	Hydrothermal method	WO_3_ nanoflowers	3 M KOH	633.3 F g^−1^ at 2 A g^−1^	89.09% after 5000 cycles	Present work

## 4. Conclusions

In summary, this study shows a simplistic synthesis of graphene oxide (GO) and tungsten trioxide (WO_3_) nanostructures, followed by hydrothermal hybridization of this transition metal oxide with carbon materials (graphene, CNTs) to produce binary and ternary nanocomposites. XRD analysis confirms the monoclinic phase and high crystallinity of WO_3_. SEM investigation demonstrates the RGO/CNT network integrated with WO_3_ nanoflowers in binary and ternary nanocomposites. Many of the vibrational modes of WO_3_ and D, G peaks of RGO/CNTs have been confirmed through Raman investigation. According to electrochemical results, the (RGO–CNT-WO_3_) electrode among all pure and binary nanocomposite electrodes has a superb specific capacitance of 633.3 F g^−1^ at a current density of 2 A g^−1^, a coulombic efficiency of 94.3% at 2 A g^−1^, and specific capacitance retention of about 89.09% for 5000 cycles at a scan rate of 1000 mV s^−1^. In short, (RGO–CNT-WO_3_) nanocomposite electrode exhibits superior specific capacitance, reversibility, cyclic stability, coulombic efficiency and low resistances making it suitable for use in supercapacitors. For high-performance supercapacitor applications, this facile methodology also provides an easy way to synthesize novel electrode materials with improved electrochemical properties.

## Conflicts of interest

There are no conflicts to declare.

## Supplementary Material

## References

[cit1] Chen J., Wang H., Deng J., Xu C., Wang Y. (2018). J. Mater. Chem. A.

[cit2] He X., Wang X., Sun B., Wan J., Wang Y., He D., Suo H., Zhao C. (2020). RSC Adv..

[cit3] Adalati R., Kumar A., Kumar Y., Chandra R. (2020). Energy Technol..

[cit4] Lemine A. S., Zagho M. M., Altahtamouni T. M., Bensalah N. (2018). Int. J. Energy Res..

[cit5] Kandasamy M., Sahoo S., Nayak S. K., Chakroborty B., Rout C. S. (2021). J. Mater. Chem. A.

[cit6] Li X.-L., Lou T.-J., Sun X.-M., Li Y.-D. (2004). Inorg. Chem..

[cit7] Cai Y., Wang Y., Deng S., Chen G., Li Q., Han B., Han R., Wang Y. (2014). Ceram. Int..

[cit8] Xiao J., Che Y., Lv B., Benedicte M.-C., Feng G., Sun T., Song C. (2021). Mater. Res..

[cit9] Tehrani F. S., Ahmadian H., Aliannezhadi M. (2020). Mater. Res. Express.

[cit10] Hariharan V., Gnanavel B., Sathiyapriya R., Aroulmoji V. (2019). Int. J. Adv. Sci. Eng..

[cit11] Peng H., Ma G., Sun K., Mu J., Luo M., Lei Z. (2014). Electrochim. Acta.

[cit12] He X., Wan J., He D., Yang X., Suo H., Zhao C. (2019). Crystals.

[cit13] Shah M. A. S. S., Khan D. I., Aziz D. M. A., Ullah P. N., Khan D. M., Adil D. S. F., Liaqat Z., Usman D. M., Tremel P. W., Tahir D. M. N. (2021). Chem.–Eur. J..

[cit14] Pragati D., Shinde A., Jun P. S. C. (2019). ChemSusChem.

[cit15] Sahoo P. K. , Tseng C.-A., Huang Y.-J. and Lee C.-P., Carbon-based nanocomposite materials for high-performance supercapacitors, in Novel Nanomaterials, ed. K. Krishnamoorthy, IntechOpen, 2021

[cit16] Wang Y., Zhang L., Hou H., Xu W., Duan G., He S., Liu K., Jiang S. (2021). J. Mater. Sci..

[cit17] Peng H., Ma G., Sun K., Mu J., Luo M., Lei Z. (2014). Electrochim. Acta.

[cit18] Xue D., Zhu D., Duan H., Wang Z., Lv Y., Xiong W., Li L., Liu M., Gan L. (2019). Chem. Commun..

[cit19] Zheng X., Miao L., Song Z., Du W., Zhu D., Lv Y., Li L., Gan L., Liu M. (2022). J. Mater. Chem. A.

[cit20] Shinde P. A., Seo Y., Ray C., Jun S. C. (2019). Electrochim. Acta.

[cit21] Ahmadian H., Tehrani F. S., Aliannezhadi M. (2019). Mater. Res. Express.

[cit22] Cai Y. W. Y., Deng S., Chen G., Li Q., Han B., Han R., Wang Y. (2014). Ceram. Int..

[cit23] Wang Y. Y. J.-G., Huang Z.-H., Kang F. (2013). Mater. Chem. Phys..

[cit24] Wang W., How Q., Lei W., Xia X., Wang X. (2014). J. Power Sources.

[cit25] Hatel R., Batoul M. (2019). J. Phys.: Conf. Ser..

[cit26] Susanti D., Haryo S., Nisfu H., Nugroho E. P., Purwaningsih H., Kusuma G. E. (2012). Front. Chem. Sci. Eng..

[cit27] Liu B., Cai D., Liu Y., Wang D., Wang L., Wang Y. (2014). Sens. Actuators, B.

[cit28] Jo E. H., Kim S. K., Chang H., Lee C., Choi S. R., Choi J. h. (2018). Aerosol Air Qual. Res..

[cit29] Gupta S. P., Nishad H. H., Patil V. B., Chakane S. D., More M. A., Late D. J., Walke P. S. (2020). Mater. Adv..

[cit30] Peng H., Ma G., Sun K., Mu J., Luo M., Lei Z. (2014). Electrochim. Acta.

[cit31] Jeevitha G., Abhinayaa R., Mangalaraj D., Ponpandian N., Meena P., Mounasamy V. (2019). Nanoscale Adv..

[cit32] Zhi M., Huang W., Shi Q., Wang M., Wang Q. (2016). RCS Adv..

[cit33] Djaoued Y., Balaji S., Bruning R. (2012). J. Nanomater..

[cit34] Manciu F. S., Enriquez J. L., Durrer W. G., Yun Y. (2010). J. Mater. Res..

[cit35] Gao L., Wang X., Xie Z., Song W., Wang L., Wu X., Qu F., Chen D., Shen G. (2013). J. Mater. Chem..

[cit36] Xing L. L., Hunag K. J., Fang L. X. (2016). Dalton Trans..

[cit37] Lu X., Zhai T., Zhang X., Shen Y., Yuan L., Hu B., Gong L., Chen J., Gao Y., Zhou J., Tong Y., Wang Z. L. (2012). Adv. Mater..

[cit38] Yuan C., Lin H., Lu H., Xing E., Zhang Y., Xie B. (2015). Mater. Lett..

[cit39] Xu J., Ding T., Wang J., Zhang J., Wang S., Chen C., Fang Y., Wu Z., Huo K., Dai J. (2015). Electrochim. Acta.

[cit40] Cai Y., Wang Y., Deng S., Chen G., Li Q., Han B., Han R., Wang Y. (2014). Ceram. Int..

[cit41] Yao S., Qu F., Wang G., Wu X. (2017). J. Alloys Compd..

[cit42] Peng W.-C., Wang S.-B., Li X.-Y. (2016). Sep. Purif. Technol..

[cit43] Nayak A. K., Das A. K., Pradhan D. (2017). ACS Sustainable Chem. Eng..

[cit44] Di J., Xu H., Gai X., Yang R., Zheng H. (2019). One-step solvothermal synthesis of feather duster-like CNT@WO_3_ as high-performance electrode for supercapacitor. Mater. Lett..

[cit45] Yang F., Jia J., Mi R., Liu X., Fu Z., Wang C., Liu X., Tang Y. (2018). Front. Chem..

[cit46] Ashraf M., Shah S. S., Khan I., Aziz M. A., Ullah N., Khan M., Adil S. F., Liaqat Z., Usman M., Tremel W., Tahir M. N. (2019). Chem.–Eur. J..

[cit47] Samal R., Chakraborty B., Saxena M., Late D. J., Rout C. S. (2019). ACS Sustainable Chem. Eng..

